# Data Report: Perceptions about gender-based violence in Latin America 2018 and 2023

**DOI:** 10.3389/fpsyt.2026.1758551

**Published:** 2026-01-23

**Authors:** Claudia Sámano-Robles, Lizeth Ramon-Jaramillo

**Affiliations:** Division of Development Studies, Center for Research and Teaching in Economics (CIDE), Mexico City, Mexico

**Keywords:** ecological model, gender-based violence, government trust, Latin-American, Latinobarómetro, violence

## 1 Introduction

This data report presents the potential use of a publicly available dataset to analyze perceptions of gender-based violence (GBV), general violence, and trust in government in 18 Latin American countries, using data from the 2018 and 2023 waves of Latinobarómetro. The dataset consolidates and standardizes key indicators that allow researchers to explore how individual perceptions of GBV relate to broader feelings of insecurity and institutional confidence in the region.

Although Latinobarómetro is not originally designed to measure gender-based violence (1, 2), we identify and select variables that can contribute to the study of violence from a structural and ecological perspective. Particularly, the construction and selection of the variables is informed by the Social Ecological Model of violence, which conceptualizes GBV as the outcome of interacting factors operating across different levels of the social environment—individual, interpersonal, community, and societal (3).

Within this framework, perceptions of GBV captured in the survey represent individual-level evaluations, but they are deeply shaped by macro-level environments. These environments include the broader climate of generalized violence (community level) (4) and the degree of confidence in governmental institutions (social/macro level) (3). By integrating variables that correspond to multiple layers of the ecological model, the dataset enables researchers to examine how perceptions of violence, insecurity, and institutional legitimacy co-evolve within and across countries, and how macro-structural conditions influence individuals’ assessments of gender-based violence.

Using comparable microdata from 2018 and 2023 makes it possible to document regional changes during a period marked by political instability, rising insecurity, increasing distrust in state institutions, and post-pandemic social transformations, including documented increases in gender-based violence across several Latin American countries during the COVID-19 pandemic (5). Although this data report does not present findings from specific empirical studies, it illustrates the analytical potential of these harmonized variables for descriptive, comparative, and ecological analyses grounded in a theoretically robust framework.

## 2 Methods

### 2.1 Data sources

The dataset is constructed from the publicly available microdata of the Latinobarómetro surveys for the years 2018 and 2023, which cover 18 Latin American countries (1, 6). Both survey waves were downloaded from the Latinobarómetro official repository and include nationally representative samples of the adult population in each country. The 2023 release is the most recent wave, published in October 2023.

### 2.2 Data collection and sample design

The 2018 wave was conducted between May and June 2018, while the 2023 wave was carried out between February and April 2023. Fieldwork in both years involved face-to-face interviews conducted by trained enumerators using standardized questionnaires.

The survey sample includes adults aged 18 and older in Latin American countries, selected through a multistage, stratified sampling design to ensure national representativeness in each country. Survey weights provided by Latinobarómetro are incorporated to adjust for sample design and response patterns. These survey weights correct for unequal probabilities of selection arising from the multistage, stratified sampling design and adjust for differential non-response across population subgroups. Their use is essential to ensure that estimates accurately reflect the national adult population in each country and that cross-country and cross-year comparisons are not biased by sample composition.

### 2.3 Variables included in the dataset

The selection and organization of variables in this dataset are guided by the Social Ecological Model of violence, a framework widely used in the study of gender-based violence to conceptualize violence as the outcome of interacting factors operating across multiple levels of the social environment (3). Rather than focusing solely on individual attitudes or experiences, this model emphasizes how individual-level perceptions are shaped by relational, community, and societal contexts, including norms, institutional arrangements, and broader conditions of insecurity.

This framework is particularly appropriate for the present dataset because Latinobarómetro does not directly measure experiences of gender-based violence but captures perceptions, fears, and institutional trust that are embedded in wider social and political environments. By mapping survey variables onto different ecological levels—individual perceptions of GBV, community-level perceptions of general violence, and macro-level confidence in government—the dataset enables researchers to explore how these dimensions co-exist and interact within and across countries.

1. Perception of gender-based violence (GBV)

Ecological level: *Individual Level (Intrapersonal), shaped by Social/Macrosystem conditions*

Individual-level variables capture whether respondents consider GBV to be a serious problem in their country and whether they perceive the situation to be worsening or improving. Although measured at the individual level, these perceptions reflect broader cultural norms and societal attitudes toward gender and violence, placing them at the intersection of the individual and macrosystem levels of the ecological model.

2. Perception of general violence and fear of victimization

Ecological level: *Community Level (Mesosystem/Exosystem), with links to the Social/Macrosystem*

Both individual-level and country-level indicators measure concern about crime, perceptions of insecurity in daily life, and the perceived likelihood of becoming a victim of violent crime. These variables capture conditions in the community and local environment, such as urban safety, crime prevalence, and exposure to violence in public spaces. At the same time, broader national conditions—such as political instability and generalized insecurity—connect this dimension to the macrosystem.

3. Trust in government

Ecological level: *Social/Macrosystem (and Political/Organizational Level in extended models)*

Individual-level measures assessing respondents’ confidence in national government institutions. This indicator captures the institutional and political environment in which perceptions of violence are formed, including expectations about government effectiveness, rule of law, and institutional legitimacy. Trust in government belongs to the outermost layer of the ecological model, shaping the cultural and structural context in which GBV and general violence perceptions arise.

### 2.4 Data processing

Several steps were undertaken to prepare the dataset:

1. Harmonization across survey wavesVariable names, response categories, and missing-value codes were standardized across the 2018 and 2023 datasets to ensure cross-year comparability.
2. Cleaning and recodingNonresponse categories (“Don’t know”, “No answer”, “Not applicable”) were recorded as missing following Latinobarómetro documentation. For ordinal questions (e.g., fear of crime), response scales were aligned to a uniform 1–4 structure.
3. Binary indicator of high concern about GBVThis variable equals 1 when respondents report that GBV is a “very serious” problem in their country, and 0 otherwise. This measure allows researchers to compare levels of perceived GBV severity consistently across countries and survey years.
4. Four-category ordinal measure of fear of violent victimizationRespondents’ self-reported fear of becoming a victim of violent crime was harmonized into a four-point ordinal scale (1 = *not at all*, 2 = *a little*, 3 = *somewhat*, 4 = *a lot*). This indicator captures different degrees of perceived insecurity and can be analyzed at both individual and aggregate levels.
5. Trust in the governmentThe original variable on trust in government—coded with both valid values (1-4) and several negative non-response categories—was recorded to separate substantive responses from non-informative answers.- Substantive trust categories were retained as an ordinal 4-point scale (1 = *a lot*, 2 = *some*, 3 = *little*, 4 = *none*).- Non-response codes (-1 to -5) were grouped into a unified category labelled “Missing/Not applicable” for comparability.
  This harmonized ordinal indicator can be used to model the relationship between institutional trust, perceptions of GBV, and general insecurity.

Responses coded as “Don’t know” were treated as missing in order to maintain consistency with Latinobarómetro documentation and to facilitate comparability across countries and survey waves. However, we acknowledge that in the context of perception-based questions on gender-based violence, such responses may reflect meaningful uncertainty, limited awareness, or discomfort with the topic rather than random nonresponse. For this reason, the dataset preserves the original response codes, allowing researchers to reclassify or explicitly analyze “Don’t know” responses as a distinct analytical category if theoretically or empirically warranted.

Users of the dataset should be aware that excluding these responses from descriptive analyses may affect estimates in settings where the proportion of “Don’t know” answers is substantial. Future research may benefit from examining the distribution and correlates of such responses as an additional dimension of social awareness or normative ambiguity surrounding gender-based violence.

### 2.5 Weighting

Survey weights provided by Latinobarómetro were preserved to maintain national representativeness.

### 2.6 Data validation

Cross-checks were performed to ensure that weighted sample sizes match official Latinobarómetro documentation for both waves.

## 3 Data analysis

Statistical associations between variables were evaluated using chi-square (χ²) tests of independence, given the categorical and ordinal nature of the indicators derived from the Latinobarómetro survey. This approach allows for the identification of statistically significant associations between perceptions of gender-based violence and macro-level variables, such as concern about violent victimization and confidence in government. Because χ² statistics assess non-independence between variables, the results indicate whether an association exists but do not provide information on the direction or magnitude of the relationship. Associations were evaluated using country-level cross-tabulations of individual responses, applying survey weights to preserve national representativeness.

**Figure 1:**
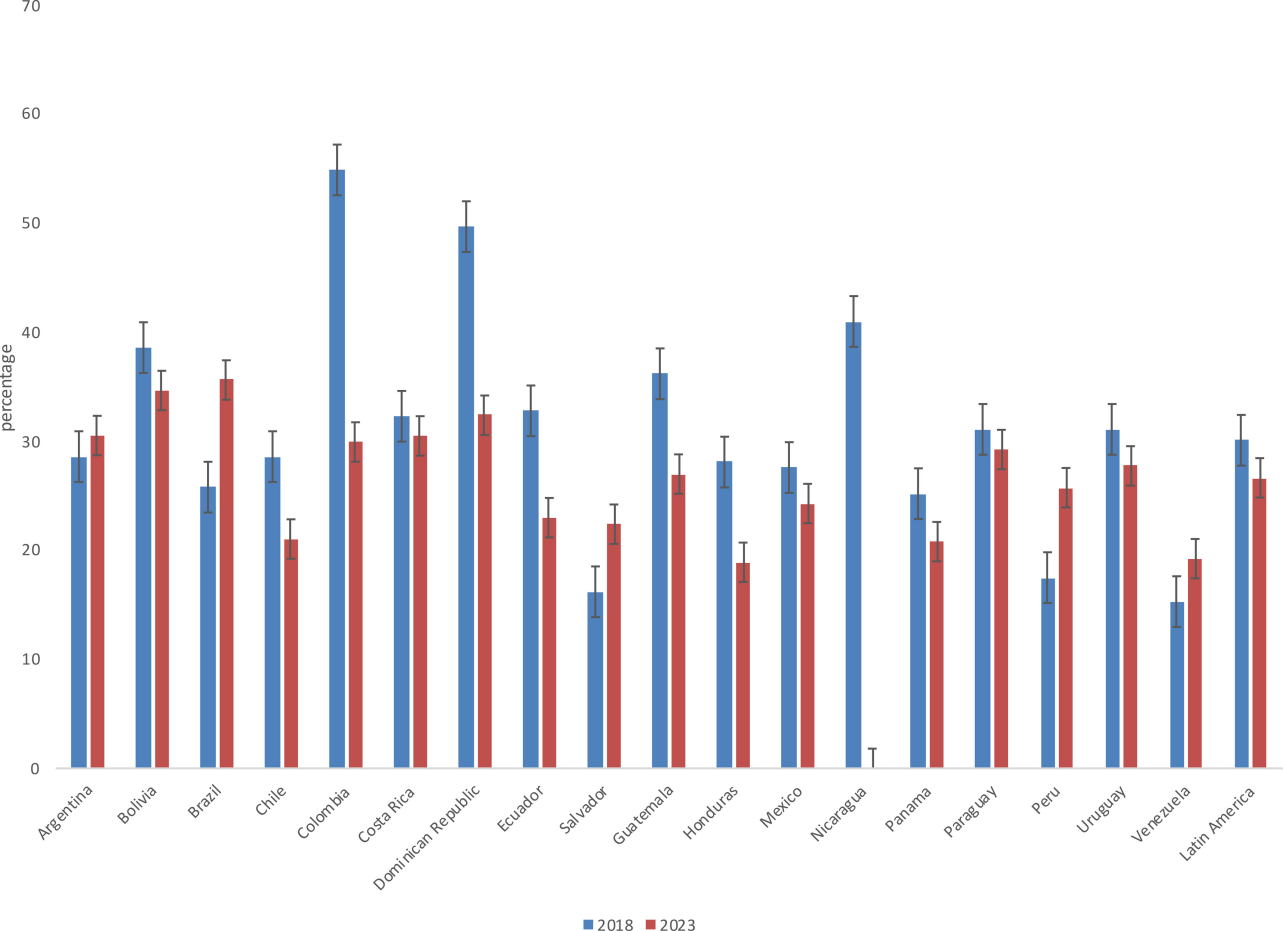
Perception about gender-based violence (GBV) as a problem by country 2018 and 2023. Source: author’s elaboration with data from 2018 and 2023.

As shown in **Figure 1**, perceptions of gender-based violence (GBV) declined in Latin America between 2018 and 2023. At the regional level, this suggests modest progress in alleviating concerns about this type of violence. However, important country-level differences are concealed by this overall trend. Notably, perceptions of domestic violence against women increased in Argentina, Brazil, El Salvador, Peru, and Venezuela during this period. Brazil, Peru, and El Salvador stand out among these countries, with respective increases of 10, 8, and 6 percentage points by 2023. Conversely, fourteen countries experienced a reduction in perceived GBV during this period. Colombia and the Dominican Republic made particularly notable progress, with decreases of 25 and 17 percentage points, respectively.

To better contextualize these trends, this report draws on the social ecological model, which distinguishes four interrelated levels of analysis: the social or macrosystem level, the community or mesosystem level, the relational or microsystem level, and the individual level. The present analysis focuses on the macrosystem level and two variables: confidence in the government and concern about becoming a victim of violent crime.

These macro-level indicators display consistent regional patterns between 2018 and 2023. In 2018, approximately 41% of individuals in Latin America believed they were likely to become victims of violent crime. This concern was particularly pronounced in Brazil (67%) and Venezuela (53%), while it was lowest in Honduras (28%). By 2023, the regional average had fallen to around 31%, representing a decline of approximately 10 percentage points. Country rankings also shifted. In 2023, Argentina (32%), the Dominican Republic (30%), and Colombia (29%) reported the highest levels of concern about victimization (1, 2).

Levels of trust in government reflect perceptions of insecurity to some extent. In 2018, 44% of Latin Americans reported having no confidence in their government. The percentage was even higher in Brazil (60%), Nicaragua (63%), and Venezuela (63%). By 2023, the regional average had decreased to 37%. Nevertheless, substantial variation persisted at the country level. For example, 62% of Peruvians, 56% of Ecuadorians, and 47% of Guatemalans reported a lack of confidence in government institutions (1, 6).

While these macrolevel factors provide valuable insight into broader patterns of violence and institutional legitimacy, it is important to analyze how they are associated with perceptions of violence against women. The results indicate that these relationships differ depending on the macro-level variable considered.

**Figure 2:**
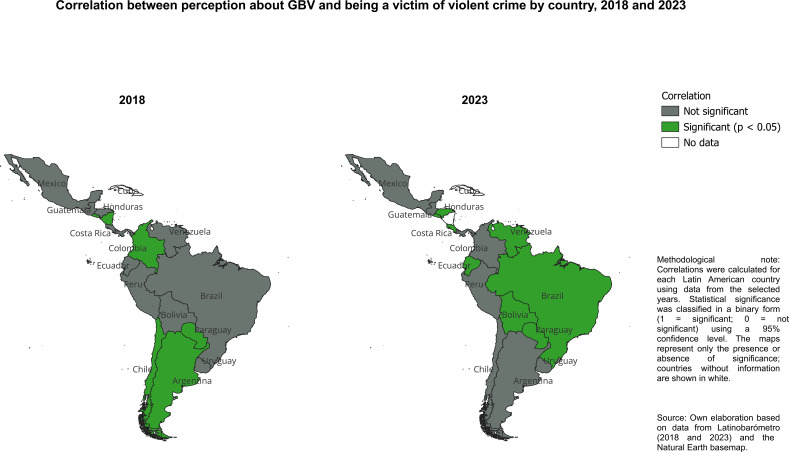
Statistical association between perception of GBV and concern about becoming a victim of violent crime by country, 2018 and 2023. Source: own elaboration with data from 1 and 2. Associations are evaluated using χ² tests of independence. Significant results indicate non-independence between variables; no direction or effect size is implied.

In 2018, the statistical association (χ² test of independence) between perceiving gender-based violence (GBV) as a serious problem and worrying about becoming a victim of violent crime was not statistically significant at the regional level in Latin America. However, this aggregate result conceals substantial cross-country heterogeneity. When the analysis is disaggregated, the association becomes statistically significant in Argentina, Chile, Colombia, El Salvador, Nicaragua, and Paraguay (see **Figure 2**). This suggests that in several national contexts, heightened perceptions of GBV are closely associated with greater concern about personal victimization. Given the use of χ² statistics, these results identify statistically significant associations but do not imply a positive or negative direction of the relationship.

By 2023, the association between these two variables had expanded geographically. In Honduras, El Salvador, Paraguay, Brazil, Venezuela, Costa Rica, Ecuador, and Bolivia, perceptions of GBV as a serious problem were significantly associated with how often individuals reported worrying about becoming victims of violent crime. Notably, only El Salvador and Paraguay exhibited statistically significant associations in both years, indicating a persistent, structural link between perceptions of gender-based violence and broader feelings of insecurity in these countries (see **Figure 2**).

Therefore, a comparison of 2018 and 2023 reveals a clear intensification and territorial expansion of the association between perceptions of gender-based violence (GBV) and concern about becoming a victim of violent crime in Latin America. While this relationship was limited to a few countries and absent at the regional level in 2018, it had become evident in a broader range of national contexts by 2023. This shift may reflect an increase in the visibility of gender-based violence, a deterioration in general security conditions, or an interaction between the two. The persistence of this relationship in El Salvador and Paraguay suggests that perceptions of GBV have become deeply intertwined with generalized fear of violent victimization in some settings.

**Figure 3:**
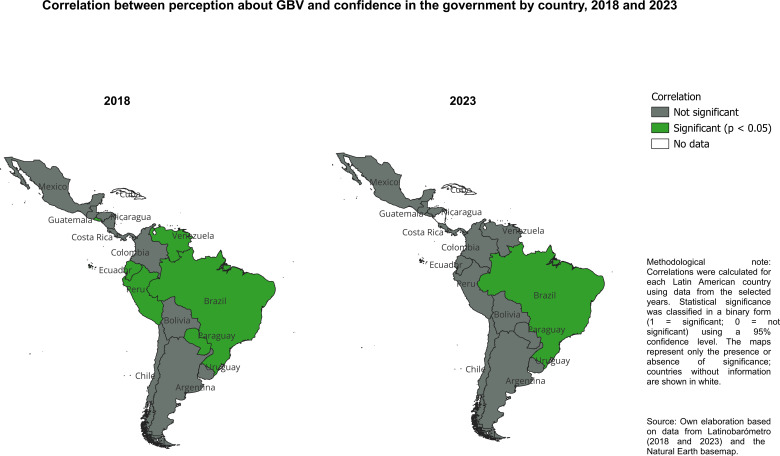
Statistical association between perception of GBV and confidence in government by country, 2018 and 2023. Source: own elaboration with data from 1 and 6. Associations are evaluated using χ² tests of independence. Significant results indicate non-independence between variables; no direction or effect size is implied.

On the other hand, the statistical association (χ² test of independence) between perceptions of gender-based violence (GBV) and confidence in the government is statistically significant at the regional level in Latin America. However, this relationship weakens considerably when examined for each country separately. In 2018, a statistically significant association was observed in only six countries: Brazil, Ecuador, El Salvador, Paraguay, Peru, and Venezuela. By 2023, this association had become even more limited. Brazil was the only country in which perceptions of GBV and confidence in the government remained significantly correlated (see **Figure 3**). This pattern suggests that although concerns about GBV may influence institutional trust overall, this effect is neither consistent nor stable over time or across national contexts. Differences in political dynamics, institutional performance, media coverage, and the prominence of GBV in policy agendas may explain why this relationship weakens or disappears at the country level.

## 4 Conclusions and final remarks

This data report introduces a harmonized, comparable dataset enabling the study of perceptions of gender-based violence, general insecurity, and trust in government across 18 Latin American countries from 2018 to 2023. Aligning key indicators with the Social Ecological Model of violence provides a structured basis for examining how macro-level conditions, such as national insecurity and institutional legitimacy, are reflected in individual-level perceptions of gender-based violence. It is important to emphasize that the indicators included in this dataset capture perceptions of gender-based violence and insecurity rather than objective prevalence or individual experiences of violence.

The descriptive analyses in this report reveal that while regional perceptions of gender-based violence (GBV) declined, this trend masks significant heterogeneity across countries. In several countries, perceptions of GBV increased during this period, and the relationship between perceived GBV and broader feelings of insecurity strengthened and became more geographically widespread by 2023. Conversely, the association between perceptions of GBV and trust in government was inconsistent, weakening at the national level and persisting in only a few cases. Observed differences between 2018 and 2023 should be interpreted with caution, as changes in perceptions of gender-based violence, general insecurity, and trust in government may reflect broader contextual shocks experienced across the region, including episodes of political instability, social unrest, and the social and institutional aftermath of the COVID-19 pandemic. These findings underscore the importance of disaggregated, country-level analyses when examining the social dynamics of violence and insecurity in Latin America.

Beyond these examples, the dataset’s main purpose is to facilitate future research. Its harmonized structure enables cross-country comparisons, assessment of temporal variation, and exploration of interactions between societal-level factors and individual attitudes. These characteristics make the dataset particularly valuable for scholars interested in regional trends, comparative perspectives, and the structural determinants of perceptions of violence.

As with any survey-based resource, however, certain limitations must be acknowledged. These include the subjectivity of perception-based measures, differences in national contexts, and the non-response issues inherent in public opinion data. Nevertheless, the regional coverage, methodological consistency, and public accessibility of the Latinobarómetro surveys provide a robust foundation for further empirical research on gender-based violence, insecurity, and institutional trust in Latin America.

The complete dataset and its documentation are publicly available through the Latinobarómetro repository, which supports transparency, reproducibility, and the development of additional methodological and analytical contributions. Ultimately, this data report illustrates how publicly available survey data can deepen our understanding of the evolving relationship between gender-based violence, social insecurity, and institutional confidence in the region.

## Data Availability

Publicly available datasets were analyzed in this study. This data can be found here: https://www.latinobarometro.org/latinobarometro-2023, https://www.latinobarometro.org/latinobarometro-2018.
